# Proof of Concept for Tumor Mutational Burden Prediction Through Biophysical Analysis Based on UHF-Dielectrophoresis

**DOI:** 10.3390/bios16030134

**Published:** 2026-02-25

**Authors:** Héloïse Daverat, Nina Blasco, Sandrine Robert, Amandine Rovini, Claire Dalmay, Fabrice Lalloué, Arnaud Pothier, Karine Durand, Thomas Naves

**Affiliations:** 1 UMR INSERM 1308, CAPTuR, University of Limoges, 2 Rue du Docteur Marcland, 87025 Limoges, France; heloise.daverat@unilim.fr (H.D.); sandrine.robert@unilim.fr (S.R.); amandine.rovini@unilim.fr (A.R.); fabrice.lalloue@unilim.fr (F.L.); 2 XLIM-UMR 7252, University of Limoges/CNRS, 123 Avenue Albert Thomas, 87060 Limoges, France; nina.blasco@xlim.fr (N.B.); claire.dalmay@xlim.fr (C.D.); arnaud.pothier@xlim.fr (A.P.); 3 Experimental Pneumology Biorescan, University of Limoges-Partnership Foundation, 2 Rue du Docteur Marcland, 87025 Limoges, France; 4 Molecular Genetics Platform of Solid Cancers, Department of Pathological Anatomy, Limoges University Hospital, 2 Avenue Martin Luther King, 87042 Limoges, France; 5 Functional Unit of Support to Translational Research and Innovation in Solid Oncology, Limoges University Hospital, 2 Avenue Martin Luther King, 87042 Limoges, France

**Keywords:** immunotherapy eligibility, tumor mutational burden, UHF-Dielectrophoresis sensing, electromagnetic signature, predictive marker

## Abstract

Tumor Mutational Burden (TMB) is a critical biomarker used to determine patient eligibility for immunotherapy with immune checkpoint inhibitors. However, its gold-standard assessment via whole exome sequencing is limited by high costs, technical complexity, and lengthy processing times. To address these challenges, we investigated whether Ultra-High-Frequency (UHF) electromagnetic wave sensing could serve as an alternative method for evaluating TMB. We analyzed the dielectrophoresis crossover frequency spectrum and corresponding electromagnetic signature (EMS) of cancer cells using a lab-on-a-chip biosensor that integrates microfluidics with dielectrophoresis-based electro-manipulation. Across seven solid tumor cell lines exhibiting diverse TMB levels, EMS exhibited an upward shift correlated with higher TMB, suggesting a relationship between mutational load and electromagnetic behavior. To further explore this connection, we artificially increased the somatic variant burden by exposing cells to the mutagen N-ethyl-N-nitrosourea (ENU). EMS measurements reliably detected the induced increase in variant load in ENU-treated cells. Overall, these findings demonstrate that EMS can detect both intrinsic TMB differences and experimentally induced increases in mutational burden, enabling refined categorization of cancer cells. Although further validation is required, this work lays the foundation for developing complementary, rapid, and accessible tools to support cancer cell stratification and guide immunotherapy decision-making.

## 1. Introduction

Over the past two decades, immune checkpoint inhibitors (ICI), such as anti-PD-1 (Programmed cell Death protein 1), anti-PD-L1 (Programmed Death-Ligand 1), and anti-CTLA4 (cytotoxic T-lymphocyte-associated protein 4) agents, have transformed the therapeutic landscape of cancer by providing durable responses and improved tolerability [1,2,3,4]. These therapies have become central to the treatment of certain cancers, especially for patients whose tumors exhibit high PD-L1 expression [5,6]. However, although PD-L1 is a relevant biomarker for predicting ICI response, patients with low PD-L1 expression may also respond favorably to these therapies [7,8,9,10]. Notably, the FDA-approved (Food and Drug Administration) monoclonal antibody pembrolizumab (Keytruda^®^), which targets PD-1, has demonstrated efficacy in patients with low PD-L1 expression [11]. Conversely, up to 15–40% of patients may fail to respond to immunotherapy despite high PD-L1 expression [12]. While multiple mechanisms may contribute to this immunotolerance [13], they remain largely unexplained. This variability in response underscores the urgent need for more accurate, robust, and comprehensive biomarkers to better guide ICI-based treatment decisions [14]. Consequently, other parameters must be considered, such as tumor mutational burden (TMB), which is a better predictor of response to immunotherapy than checkpoint inhibitor expression alone.

Indeed, TMB has garnered significant interest in recent years, with an exponential increase in data and publications in the literature. Specifically, TMB has emerged as a critical biomarker for predicting response to immunotherapy [15,16,17,18]. TMB quantifies the total number of non-synonymous somatic mutations and additional coding variants per megabase within the coding regions of a tumor genome. Tumors with high TMB, defined as ≥10 mutations per megabase (Mut/Mb), are more likely to respond to immunotherapy [19,20,21,22]. It is important to note that individual mutations vary in their functional impact, with some mutations more likely to alter cellular behavior, signaling pathways, and therapeutic response. Because mutations generate neoantigens or tumor-specific antigens recognized by the immune system as foreign, an anti-tumor response is triggered when ICI release the natural brakes on the immune system [17,23]. Therefore, an accurate assessment of TMB can aid in selecting patients most likely to benefit from ICI. However, despite its potential, TMB measurement using the current gold standard, whole exome sequencing (WES), is often costly, resource-intensive, and time-consuming for routine clinical application [24]. This limitation restricts its integration into standard oncological workflows, particularly in resource-constrained settings. To address this, a novel technology based on Dielectrophoresis (DEP) could provide a rapid method for identifying mutation rates.

DEP is a label-free electrokinetic technique that uses electric fields to manipulate and sort particles based on their intrinsic dielectric properties. DEP can also be employed to characterize specific physical specificities of biological cells. Biologically, cells behave as polarizable dielectric entities. Variations in their electrical permittivity and conductivity across different frequencies reflect their unique cellular characteristics, summarized as distinctive bioelectric signatures [25]. Depending on the frequency range of the electric field interacting with the cell, these signatures highlight different cellular components. At low frequencies (<10 kHz), they primarily represent the properties of the cell membrane, whereas at higher frequencies (100 kHz–500 MHz), they provide information about internal structures such as the nucleus, organelles, and cytoplasmic contents [26,27]. Operating in the ultra-high frequency (UHF) band, electromagnetic waves can penetrate the plasma membrane and reach the cell’s interior without causing damage. Consequently, ultra-high frequency dielectrophoresis (UHF-DEP) enables intracellular dielectric characterization while preserving cell viability, facilitating further investigations.

A dielectrophoresis experiment on a cell subpopulation may involve measuring the UHF-DEP crossover frequencies (CF) for a representative subset of cells from the sample. Specifically, when a cell is exposed to a non-uniform electric field, a crossover frequency is observed at the point where the field’s frequency is adjusted such that the cell shifts from a negative DEP regime (cell repulsion) to a positive DEP regime (cell attraction) [26]. At this characteristic frequency, the DEP force becomes zero. This DEP crossover at UHF is can be detected using a quadrupole microelectrode structure, as demonstrated in previous studies [28,29]. In practice, the CF value varies among individual cells within the studied subpopulation due to inherent cellular heterogeneity, resulting in variable dispersion of these characteristic frequencies. Once characterized by a representative number of cells, a median crossover frequency value is calculated. The electromagnetic signature (EMS) of the population is defined as the interquartile frequency range around the CF median value, encompassing ±25% of all measured cells.

Ultra-High-Frequency electromagnetic sensing presents as a novel approach for assessing the mutational load by analyzing the dielectrophoretic properties of cancer cells within the tens of MHz range. Although UHF-DEP does not directly measure mutational burden, it is plausible that UHF-DEP can detect electromagnetic properties indirectly associated with the cell’s mutational status. Mutations alter DNA sequences, leading to changes in genome and chromatin structure, disrupting gene expression, and causing modifications in the genes, RNAs, and proteins they encode. Notably, non-synonymous mutations result in either loss or gain of protein function, triggering significant changes in cellular phenotype, homeostasis, and behavior. These mutations can affect cell signaling pathways, metabolic processes, and cell cycle regulation—key hallmarks of cancer cells [30].

Here, we propose a model in which EMS of cells, measured by UHF-DEP, can indirectly provide insights into the genomic characteristics of cancer cells as a complementary biophysical parameter. This study potentially paves the way for exploring the relationships among between genotype, phenotype, and biophysical properties, suggesting that EMS data could help to guide patient eligibility for ICI treatments. Our findings highlight the potential of EMS to identify high-TMB solid tumor cell lines and to detect experimentally induced increases in somatic variant load. Future research will focus on validating the correlation between EMS and mutational burden across a broader range of cancer cell lines and tumors, including matched healthy counterparts, to reinforce EMS’ role as a predictive biomarker in oncology.

## 2. Materials and Methods

### 2.1. Cell Culture

Six solid cancer cell lines were obtained from the American Type Culture Collection (ATCC): lung adenocarcinoma (A549, H1975), glioblastoma (U87-MG), melanoma (SK-MEL-5 [MEL-5], SK-MEL-28 [MEL-28]) and, colon adenocarcinoma (SW480). The medulloblastoma cell line DAOY was kindly provided by the Dr. Mirella Tanori from the Italian National Agency for New Technologies, Energy and Sustainable Economic Development. All cell lines were cultured under adherent conditions in Dulbecco’s Modified Eagle Medium (DMEM) GlutaMAX (Gibco, Thermo Fisher Scientific, Illkirch, France). The medium was supplemented with 10% fetal bovine serum (FBS) (IDbio, Limoges, France) and 1% antibiotics (penicillin and streptomycin at 100 U/mL and 100 μg/mL, respectively) (Gibco, Thermo Fisher Scientific, Illkirch, France). All cell cultures were maintained at 37 °C in a humidified atmosphere with 5% CO_2_. Cells were cryopreserved by freezing at −80 °C in complete medium containing 10% Dimethylsulfoxide (DMSO) (Sigma-Aldrich, Saint-Quentin-Fallavier, France). To ensure experimental integrity, we performed weekly PCR-based screening of all cell cultures for mycoplasma contamination.

### 2.2. ENU Treatment

H1975 and U87-MG cell lines were exposed to 100 μM of the mutagenic agent N-ethyl-N-nitrosourea (ENU, Sigma-Aldrich, Saint-Quentin-Fallavier, France) for 24 h to increase their mutational load, thereby raising the number of somatic variants. This treatment was repeated six times at one-week intervals, allowing sufficient time for the clearance of cells undergoing death due to treatment-induced genotoxicity and for the enrichment of viable cell population.

### 2.3. Electromagnetic Signature Analysis

**Figure 1:**
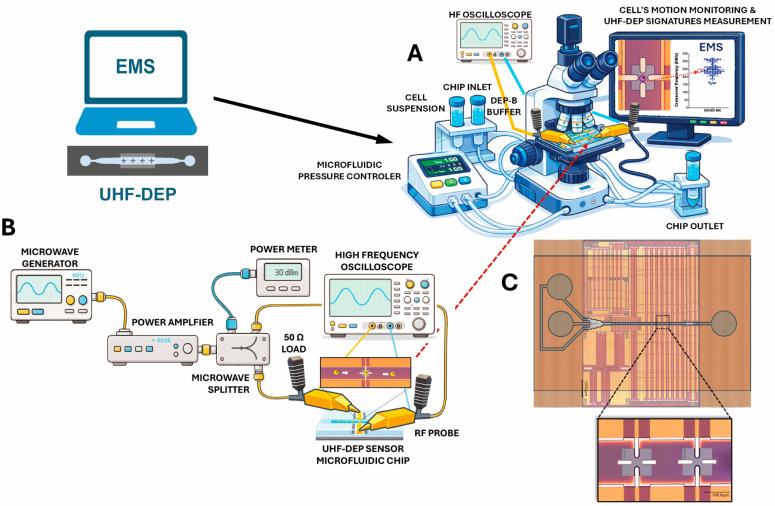
(**A**) Schematic view of the implemented setup for measuring crossover frequencies in microfluidic environment to establish the cell’s electromagnetic signature (EMS). (**B**) Focus on the UHF DEP signal generation and its application to the quadrupole electrode DEP sensor. In practice, microwave generation was performed using an Rohde & Schwarz SMB100A source (Rohde & Schwarz, Munich, Germany). The delivered power was monitored and adjusted with an Rohde & Schwarz NRP8S diode power sensor (Rohde & Schwarz, Munich, Germany), enabling high-accuracy feedback control across the 10 MHz to 1 GHz range. A Bonn Elektronik GmbH BLWA 110-5M wideband power amplifier (Bonn Elektronik GmbH, Holzkirchen, Germay) was used to achieve a DEP signal magnitude of 3.5 to 4 VRMS on the sensor’s electrodes. A Pulsar PS2-26-450/13S MW splitter (Pulsar, Clifton, NJ, USA) allowed simultaneous in-phase biasing of the north and south quadrupole electrodes, while the other were grounded. The sensors were biased on-wafer via medium-power MPI TITAN T26P-GSG-150 RF probes (MPI, Hsinchu, Taïwan); by matching these to a 50 Ω load, we minimized voltage standing wave ratio (VSWR) effects and ensured a consistent signal level across all frequencies, despite the sensor’s high impedance. A wideband Tektronix DPO4104 oscilloscope (Tektronix, Beaverton, OR, USA) was used to verify the quality of the applied waveform. An Elveflow OB1 MK3 controller (Elveflow, Paris, France) managed cell fluidics by pressurizing the channel inlet relative to the outlet, allowing fine-tuned control of flow rates and cell speed, thereby guiding the samples directly to the sensing area. This complete setup and measurement principles are adapted from our previous works [28,29]. (**C**) Photograph of the microfluidic chip platform implemented under a PDMS channel, with a close-up view of the quadrupole sensors. The platform was fabricated using back-end-of-line IHP microelectronics CMOS technology as part of the SMUCASTEC project (GA No. 737164).

The electromagnetic signatures (EMS) of the target cell subpopulations of interest were established by measuring the intrinsic bioelectric characteristics of the cells using a lab-on-a-chip electromagnetic biosensor [31] capable of sensing intracellular dielectric properties (Figure 1A). This device integrates microfluidics and dielectrophoresis technologies to characterize cells in suspension through a high-frequency (>10 MHz) electromagnetic field (Figure 1A–C). An external flow controller drives the particle suspension through the microfluidic channel until the cells reach the sensing area. The UHF-DEP electric field applied to the cells is generated using a continuous-wave high-frequency signal generator combined with a power amplifier. A power splitter is used to apply the same signal, in both magnitude and phase, to the two sensor inputs. These signals are delivered to two wideband and matched to 50 Ohm impedance microstrip waveguide lines on the middle of which the micro size sensor device is implemented. RF probes are used to ensure proper connectivity with the power splitter; for which a 50 Ohm load is connected in parallel on each probe to prevent standing wave effects caused by the high impedance of the sensor. A high-speed oscilloscope monitors the DEP signal applied to the sensor in real time.

**Figure 2:**
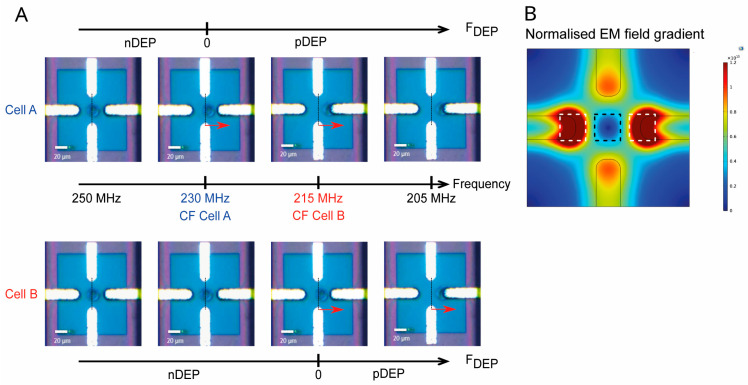
(**A**) Single-cell crossover frequency (CF) measurement using one sensor: Dielectrophoretic force (FDEP) response of cell A (blue) under an applied ultra-high frequency (UHF) signal for frequencies between 250 and 205 MHz. The CF is determined at 230 MHz, where the force transitions from negative dielectrophoresis (nDEP) to positive and attractive dielectrophoresis (pDEP). Similarly, the dielectrophoretic force response of cell B (red) is shown under the same UHF signal frequency range, with its CF measured at 215 MHz. (**B**) Numerical simulation of the electric field gradient generated by the biased quadrupole (COMSOL Multiphysics^®^ version 6.1). The color scale represents the normalized electromagnetic (EM) field gradient intensity (V^2^/m^3^), which governs the magnitude of the dielectrophoretic (DEP) force. The black square indicates the region with the weakest EM field gradient, favored by cells exhibiting nDEP behavior. The white squares indicate regions with the strongest EM field gradients, where cells are attracted by pDEP.

To measure the cell crossover frequency, cells are driven toward the four-electrode system. When a cell reaches the vicinity of the quadrupole, the flow is stopped, and a high-frequency negative DEP (nDEP) signal is applied (at 250 MHz, as shown in Figure 2A). This causes the cell to center itself in the region where the electromagnetic field gradient is weakest (black square area in Figure 2B). By gradually decreasing the frequency, a slight movement of the cell can be observed due to thermal and Brownian noise when the DEP force becomes very weak. As the frequency is further decreased until the cell switches to positive DEP (pDEP), the cell moves toward a lateral electrode (white square, Figure 2A). The frequency at which cellular movement begins is defined as the UHF dielectrophoresis CF. For cell A, the CF is 230 MHz, and for cell B, it is 215 MHz (Figure 2B). This measurement is repeated on several cells for each sample.

### 2.4. DEP Medium Preparation

The DEP medium is a specially formulated osmotic solution designed for electro-manipulation. It consists of deionized water supplemented with precisely controlled amounts of sucrose (8.5%), Tris base (1 mM), and anhydrous MgCl_2_ (0.69 mM) (Sigma-Aldrich, Saint-Quentin-Fallavier, France). To ensure compatibility with live-cell experiments, the medium’s pH was adjusted to the physiological value of 7.4. The conductivity of the medium was carefully controlled and monitored at 20 mS/m before each experiment using a calibrated conductivity meter. All measurements were conducted within a narrow conductivity range to ensure reproducibility, consistent with previously reported DEP protocols [28,29,32].

### 2.5. Genomic DNA Extraction and Qualification

Genomic DNA was extracted using the Maxwell^®^ CSC Genomic DNA Kit for cell lines on the Maxwell^®^ CSC Instrument PLC (Promega Corporation, Madison, WI, USA). DNA concentrations were measured with the Qubit™ Broad-Range (BR) dsDNA Assay Kit (Thermo Fisher Scientific, Illkirch, France) using a Qubit™ fluorometer (Thermo Fisher Scientific, Illkirch, France).

### 2.6. Tumor Samples

Genomic DNA from patient breast tumor samples for TMB analysis was obtained from the Centre de Ressources Biologiques (CRBioLim), in strict compliance with current regulations governing the use of biological samples for research purposes. The research was approved by the Institutional Review Board (IRB) of the Anatomo-Pathology Department of Dupuytren Hospital under approval number 2022-029. All procedures were conducted in accordance with the ethical standards of the Declaration of Helsinki. Written informed consent was obtained from all participants prior to their inclusion in the study.

### 2.7. TMB Determination by Next-Generation-Sequencing

TMB measurement was performed using 20 ng of genomic DNA with the Oncomine™ Tumor Mutation Load Assay kit (Thermo Fisher Scientific, Illkirch, France). Library quantification was conducted using the Ion Library TaqMan™ Quantification kit (Thermo Fisher Scientific, Illkirch, France) on the QuantStudio 5 real-time quantitative PCR instrument (Thermo Fisher Scientific, Illkirch, France). Libraries were loaded onto an Ion 540 chip using the Ion Chef™ Instrument (Thermo Fisher Scientific, Illkirch, France) sequencing array preparation system and subsequently sequenced on the Ion S5™ instrument (Thermo Fisher Scientific, Illkirch, France). Data analysis was performed using the online Ion Reporter™ Software version 5.20 (Thermo Fisher Scientific, Illkirch, France).

### 2.8. Statistical Analysis

Statistical analyses were conducted using GraphPad Prism software (version 10.0, Dotmatics, San Diego, CA, USA). Non-parametric Mann–Whitney tests were applied, and Pearson correlation analyses were used to assess linear relationships. Differences were considered statistically significant when *p* < 0.05. All experiments were independently repeated at least three times to ensure robustness and reproducibility. For all statistical analyses, a minimum of 200 individual cells per condition were collected from at least three independent biological replicates. Individual cell measurements were aggregated within each replicate, and the resulting replicate means were used as the unit of analysis.

## 3. Results

### 3.1. Analysis Workflow Development: Focus on Maintaining Cell Viability

**Figure 3:**
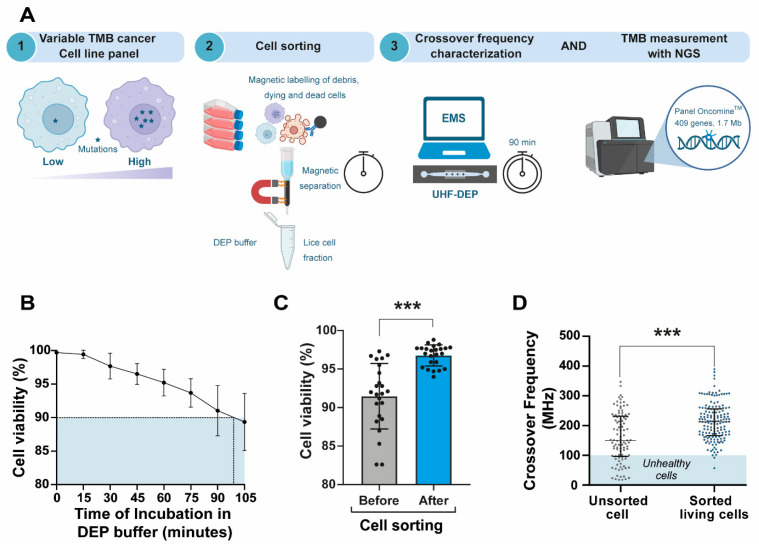
Analysis workflow for ensuring cell viability in reliable EMS and TMB assessment. (**A**) Workflow for assessing EMS and TMB in cancer cell lines. This schematic illustrates a three-step analysis process. Step 1 involves selecting a panel of cancer cell lines panel characterized by variable TMB levels, ranging from low to high. Step 2 details the sample preparation process, where cells are consistently cultured and prepared using standardized and normalized methods. This includes magnetic labeling and cell sorting, resulting in a live cell fraction suspended in DEP-B. Step 3 depicts the characterization of CF using UHF-DEP, conducted over a 90 min period following the initiation of DEP-B addition. TMB measurement is performed using the Oncomine™ targeted NGS panel, with the mutational load calculated by normalizing non-synonymous somatic mutations against the total exonic bases with sufficient coverage. (**B**) Measurement of cell viability in H1975 cells incubated in DEP-B over time post-sorting, from 0 to 100 min (n = 7). The blue shaded area indicates the critical viability threshold set at 90%. (**C**) Improved viability of the cell population post-sorting. The scatter dot plot with bar illustrates the percentages of viable cells in the population before and after sorting across a panel of cancer cell lines (H1975, A549, MEL5, MEL28, SW480, and U87-MG), with a total of 30 samples analyzed. Data are presented as mean values ± standard deviation (SD). The enhancement in cell viability following sorting was statistically significant, as determined by an unpaired *t*-test (*** *p* < 0.0001). (**D**) CF of H1975 sorted living cells compared to unsorted cells. The scatter dot plot displays individual CF measurements in MHz for sorted living cells (N = 157) and unsorted cells (N = 102). The enrichment of viable cells significantly increased the EMS frequency range, as determined by the Mann–Whitney test (*** *p* < 0.0001). The shaded area indicates the range of CF for unhealthy cells.

Although EMS depends on several factors, the buffer used, DEP-B (DEP-Buffer), is compatible with both the conductivity (anionic) required for EMS analysis and the physiological osmolarity needed to minimize cell toxicity. Molecular and cellular alterations in dying cells can otherwise introduce significant artifacts, including (i) changes in cell structure and (ii) loss of DNA integrity following membrane disruption. Because cell viability is a critical parameter influencing these measurements, a systematic three-step cell sorting process (Figure 3A, step 2) was incorporated into the workflow designed to analyze TMB and EMS from cell lines with varying TMB levels (Figure 3A, steps 1 to 3). Consistent with this above workflow, DEP-B preserved cell viability for up to 90 min, after which viability decreased by more than 10% (*p* < 0.0001) (Figure 3B). Using a dead cell removal assay (Supplementary Materials and Methods), we significantly increased the proportion of viable cells (*p* < 0.0001, Figure 3C) before suspending them in DEP-B. As expected, median EMS frequency values were significantly lower in unsorted cells compared to sorted live cells, likely due to the influence of cell death (*p* < 0.0001, Figure 3D). Altogether, the cell handling time after sample preparation (Figure 3A, step 2) was limited to a maximum of 90 min to ensure that cell death remained below 10% during EMS characterization (Figure 3A, step 3). The reproducibility of this method was demonstrated across multiple biological replicates, as shown in Supplementary Figure S1. These results highlight the robustness of UHF-DEP in providing consistent measurements.

Overall, this workflow preserves cell viability, allowing for accurate TMB characterization and enhancing the reliability of our analyses to better reflect the biological state of the samples.

### 3.2. Discrepancies Between Methods for TMB Calculation

**Figure 4:**
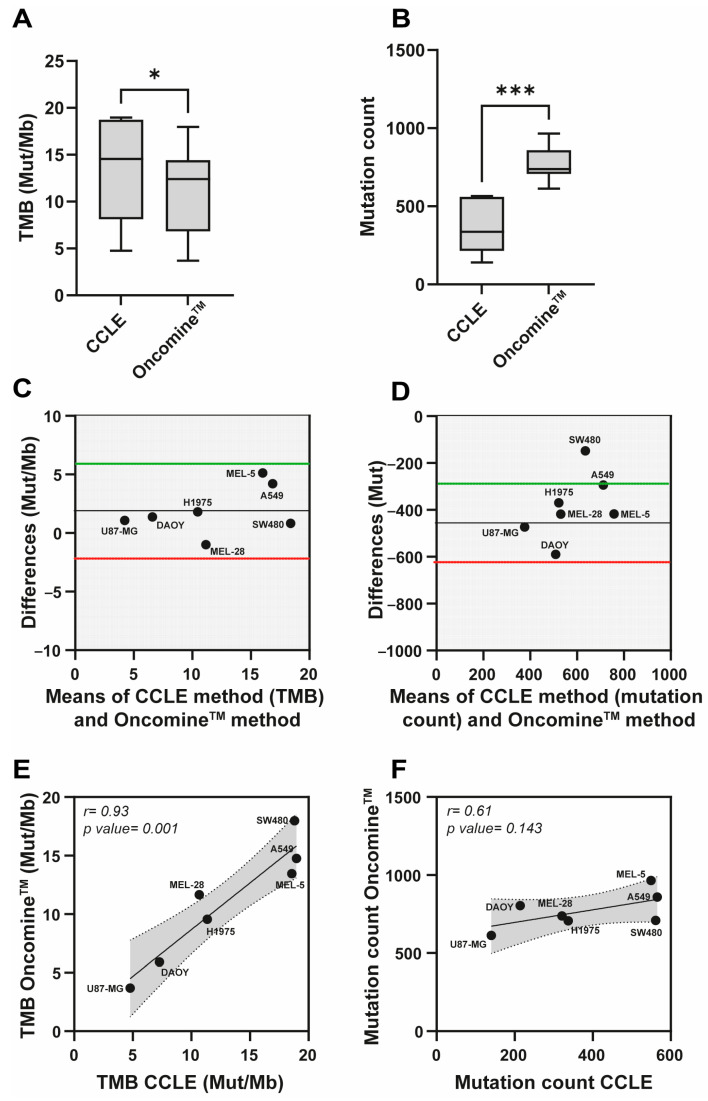
Comparison of Tumor Mutational Burden (TMB) and mutation counts between Oncomine methods and publicly available data from CCLE. (**A**) Box plot comparing TMB (Mut/Mb) values between CCLE and Oncomine, showing a statistically significant difference (*p* < 0.05, *). (**B**) Box plot comparing mutation counts between CCLE and Oncomine, indicating a highly significant difference (*p* < 0.001, ***). (**C**) Bland–Altman plot illustrating the differences in TMB (Mut/Mb) between the CCLE and Oncomine methods, plotted against the mean values for each method. The red and green horizontal dashed lines represent the mean difference and the limits of agreement, respectively. (**D**) Bland–Altman plot displaying the differences in mutation counts between the CCLE and Oncomine methods, plotted against the mean values for each method. The lines indicate the mean difference and the limits of agreement. (**E**) Correlation plot between TMB values (Mut/Mb) from the CCLE and Oncomine methods, with a Pearson correlation coefficient (*r*) of 0.93 (*p* = 0.001). (**F**) Correlation plot between mutation counts from the CCLE and Oncomine methods, showing a moderate correlation (*r* = 0.61, *p* = 0.143). In panels (**E**,**F**), dashed lines represent the 95% confidence intervals of the linear regression. The gray shaded area indicates the prediction band, reflecting the dispersion of individual samples around the fitted regression line.

To evaluate the accuracy and consistency of tumor mutational burden (TMB) and mutation count calculations, we compared the Oncomine™ oncology panel with publicly available data using a panel of seven human cancer cell lines representative of solid tumors (U87-MG, DAOY, H1975, MEL-28, MEL-5, A549, and SW480) (Figure 4A). Consequently, we curated publicly available TMB data from the Cancer Cell Line Encyclopedia (CCLE) [33], available through the Broad Institute via cBioPortal (https://www.cbioportal.org/ (accessed on 2 July 2025)) [34]. In parallel, experimental data were generated from the same cell lines: after removing dead cells, DNA was extracted and used as template for mutation analysis, ensuring that each sample met quality control standards to exclude DNA degradation. TMB scores and mutation loads were then calculated using the Oncomine™ assay and compared to the CCLE data.

The Oncomine™ assay consistently revealed lower TMB values compared to CCLE (Figure 4A), with statistically significant differences (*p* < 0.05). Specifically, the TMB ranged from 3.69 to 17.97 Mut/Mb for Oncomine™ and from 4.77 to 18.8 Mut/Mb for CCLE across the different cell lines (Table 1A). Additionally, Oncomine™ showed a consistently higher total mutation count compared to CCLE (Figure 4B), with differences that were highly significant (*p* < 0.0001), underscoring methodological differences in mutation calling. This discrepancy between Oncomine™ and CCLE highlights the variability that may arise from differences in mutation-calling algorithms, sequencing depth, variant filtering criteria, panel design, and the genomic regions considered for TMB normalization, including potential differences in the inclusion of non-coding regions. Similarly, direct comparison of TMB values obtained with the Oncomine™ assay and Foundation Medicine (FMI) on two patient tumor samples (Tumor A and Tumor B) revealed that Oncomine™ tends to underestimate TMB in Tumor B, but overestimate it in Tumor A (Table 1B). Together, these results demonstrate that variability in TMB estimation is not limited to in vitro analyses but is also evident in tumor sample comparisons (Oncomine™ vs. FMI), highlighting the ambiguity and lack of robustness of current approaches.

Bland–Altman analyses were conducted to evaluate the agreement between the Oncomine™ and CCLE methods for TMB (Mut/Mb) and mutation count. For TMB, Bland–Altman plots demonstrated strong agreement between Oncomine™ and CCLE, with data points closely aligned along the solid line, indicating a small mean difference and acceptable limits of agreement (Figure 4C).

However, greater variability was observed for mutation counts (Figure 4D), highlighting differences in how mutations are counted and included in TMB calculations across methods. A statistically significant correlation was found for TMB values between Oncomine™ and CCLE (*r* = 0.93, *p* = 0.001; Figure 4E), indicating high concordance between the two methods in assessing TMB. In contrast, the correlation for mutation counts was moderate and not statistically significant (*r* = 0.61, *p* = 0.143; Figure 4F), suggesting that methodological differences in mutation calling have a greater impact on mutation count estimates across methods.

These results highlight the challenges of comparing TMB and mutation count data obtained from different methods. Although there is high concordance in TMB between Oncomine™ and CCLE, the observed variability in mutation counts underscores the need to develop standardized methodologies to ensure consistent and reliable results. This is especially critical for the clinical application of TMB as a predictive biomarker for immunotherapy.

### 3.3. Relevance of EMS for Determining TMB in Solid Tumor Cells

**Figure 5:**
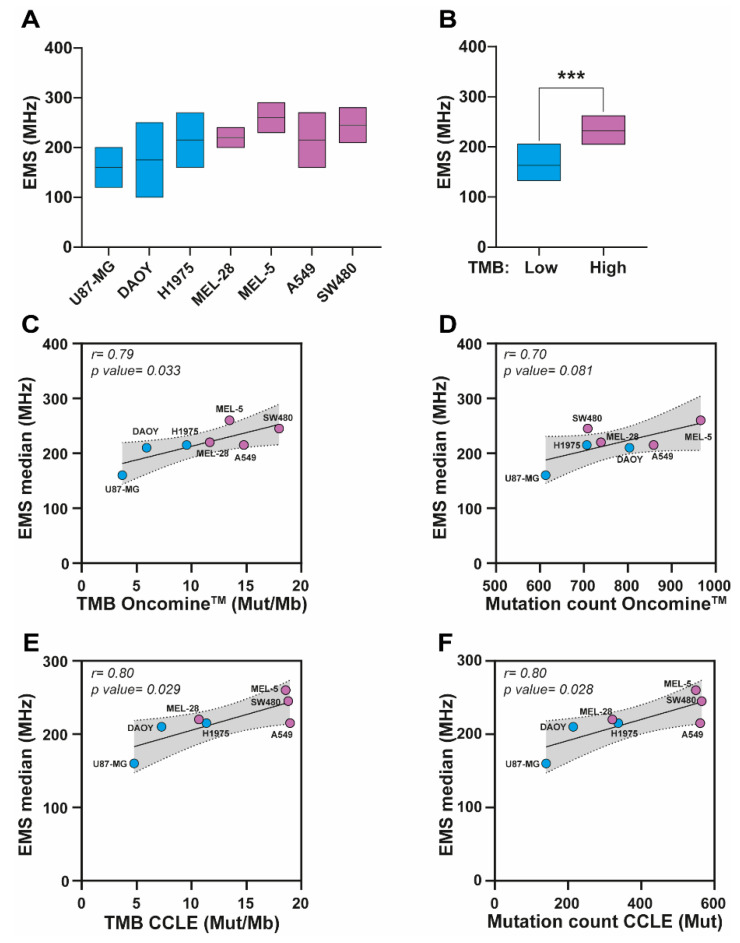
Relationship between electromagnetic signature (EMS) median values and Tumor Mutational Burden (TMB) or mutation count across the Oncomine method and publicly available data from CCLE. (**A**) Box plot showing the EMS frequency range (MHz) for various cell lines (U87-MG, DAOY, H1975, MEL-28, MEL-5, A549, and SW480). (**B**) Box plot comparing compiled raw EMS frequency range (MHz) values for cell lines with low (<10 Mut/Mb) versus high TMB values (>10 Mut/Mb). A statistically significant difference is observed between the groups (*p* < 0.001, ***). (**C**) Correlation plot between TMB (Mut/Mb) from the Oncomine method and EMS median values (MHz), showing a strong positive correlation (*r* = 0.79, *p* = 0.033). (**D**) Correlation plot between mutation count from the Oncomine platform and EMS median values (MHz), showing a moderate positive correlation (*r* = 0.70, *p* = 0.081). (**E**) Correlation plot between TMB (Mut/Mb) from CCLE publicly data and EMS median values (MHz), demonstrating a strong positive correlation (*r* = 0.80, *p* = 0.029). (**F**) Correlation plot between mutation count from the CCLE public data and EMS median values (MHz), showing a strong positive correlation (*r* = 0.80, *p* = 0.028). In panels (**C**–**F**), dashed lines represent the 95% confidence intervals of the linear regression. The gray shaded area indicates the prediction band, reflecting the dispersion of individual samples around the fitted regression line.

EMS measurements revealed that values varied across the panel of seven cancer cell lines (Figure 5A). Stratification according to TMB status showed significantly higher EMS frequency ranges in cell lines with high TMB (purple EMS box) compared to those with low TMB (blue EMS box) (*p* < 0.001; Figure 5B). To further investigate this association, we examined the relationship between EMS and TMB or mutation counts derived from the Oncomine™ assay. EMS correlated significantly with TMB (*r* = 0.79, *p* = 0.033; Figure 5C), while the correlation with mutation count showed only a non-significant trend (*r* = 0.70, *p* = 0.081; Figure 5D). We then compared these results with data from the CCLE, observing significant correlations between EMS and CCLE-derived TMB (*r* = 0.80, *p* = 0.029; Figure 5E) as well as mutation counts (*r* = 0.80, *p* = 0.028; Figure 5F).

Together, these findings indicate that EMS measurements are consistently correlated with mutational load across various datasets.

### 3.4. Assessing TMB and EMS in Response to Evolving Mutations

**Figure 6:**
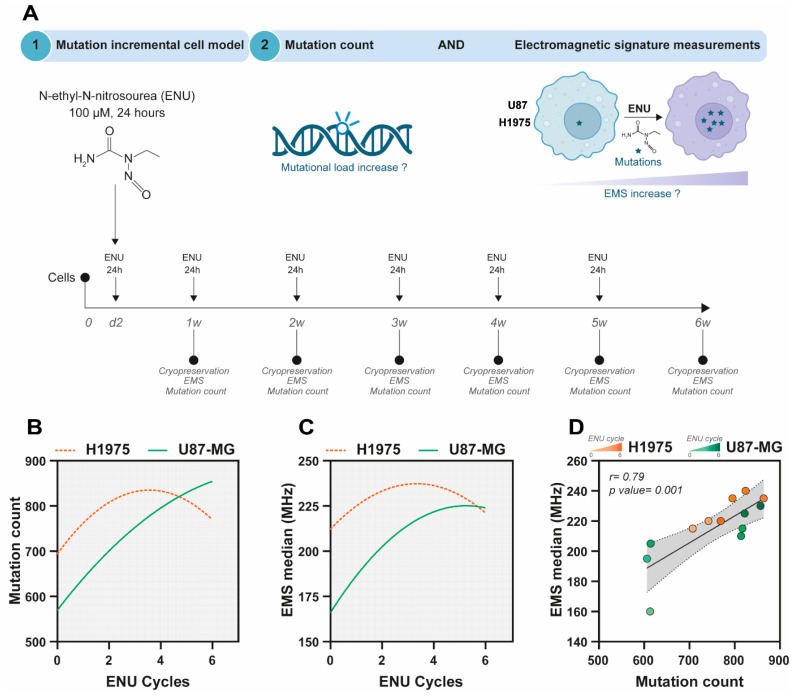
Analysis of mutation count and EMS in a mutation-incremental cell model using ENU treatment. (**A**) Schematic representation of the experimental design. Cells (U87-MG and H1975) were treated with N-ethyl-N-nitrosourea (ENU) (100 µM for 24 h) to increase mutational load. Mutations were measured at different time points (0 [control], 1, 2, 3, 4, 5, and 6 weeks) with corresponding cryopreservation, mutation count, and EMS measurement. (**B**) Mutation count over ENU cycles for both U87 and H1975 cells. (**C**) EMS median values (MHz) over ENU cycles for U87 and H1975 cells, showing a progressive increase in EMS as mutational load increases. (**D**) Correlation plot between mutation count and EMS median values (MHz) for U87 and H1975 cells, showing a strong positive correlation (*r* = 0.79, *p* = 0.001), indicating that higher mutation counts are associated with increased EMS. Dashed lines represent the 95% confidence intervals of the linear regression. The gray shaded area indicates the prediction band, reflecting the dispersion of individual samples around the fitted regression line.

To directly assess the relationship between mutation accumulation and EMS, we established an incremental mutational burden model by repeatedly exposing U87-MG and H1975 cells to N-ethyl-N-nitrosourea (ENU) (Figure 6A). It is important to note that ENU induces random mutations, most of which remain uncharacterized and have unknown clinical implications. Consequently, the TMB cannot be accurately calculated from these mutations. Instead, we rely on the mutation count as a readout, which, while informative, does not provide the same clinical insight as TMB measurements. Across successive ENU cycles, both cell lines exhibited a progressive increase in mutation counts, although the kinetics differed between models (orange dotted line: H1975, green line: U87-MG, Figure 6B). Concurrently, EMS values increased in a similar stepwise manner after several ENU cycles (orange dotted line: H1975, green line: U87-MG, Figure 6C). Correlation analysis confirmed a strong association between EMS median frequency and mutation count across the ENU exposure series (*r* = 0.79, *p* = 0.001; Figure 6D).

These results demonstrate that EMS dynamics closely track increases in mutational load in vitro, supporting the concept that EMS monitoring can serve as a predictive indicator of mutation count.

## 4. Discussion

Biomarkers, whether prognostic or predictive of treatment response, are fundamental to precision oncology and represent a major challenge in global public health. Developing new technologies that can rapidly and affordably assess these biomarkers is a central objective of translational and interdisciplinary research aimed at improving patient stratification. The advent of immune checkpoint inhibitors (ICIs) has revolutionized cancer therapy but has also underscored the urgent need for reliable biomarkers to optimize patient selection [35]. Among emerging candidates, tumor mutational burden (TMB) has received FDA approval to determine eligibility for pembrolizumab treatment [20]. However, widespread clinical adoption remains limited due to the cost, technical complexity, and turnaround time associated with whole-exome sequencing, the current gold standard for TMB quantification [24]. Furthermore, the applicability of a universal threshold of 10 mutations per megabase remains a subject of debate [36].

To address these limitations, we investigated ultra-high-frequency (UHF) electromagnetic wave analysis as a novel, label-free method to infer TMB by measuring the dielectrophoretic crossover frequencies of cancer cells. Since mutations can alter DNA sequences and consequently affect gene expression, protein function, and intracellular organization—ultimately modifying signaling, metabolism, and proliferation—these molecular alterations may influence the dielectric properties of cells [37]. Nevertheless, the transition from genomic alterations to EMS remains a significant conceptual limitation of the present study. While UHF-DEP is sensitive to intracellular heterogeneity and organelle-level organization, we do not provide a formal biophysical model directly linking specific mutation-driven structural changes to the measured dielectric response. In particular, a rigorous mechanistic interpretation would require dielectric modeling frameworks, such as multi-shell cell models, that explicitly describe the respective contributions of the plasma membrane, cytoplasm, and intracellular compartments to the overall dielectric behavior. Incorporating such models would be a critical next step to move beyond empirical correlations and quantitatively relate mutation-induced intracellular alterations to EMS measurements. Although UHF-DEP characterization does not directly quantify mutations, it offers an indirect yet functional approach to assess mutation-linked biophysical changes. This method provides several advantages: it bypasses DNA extraction and sequencing, enables faster initial screening, and is non-destructive, thereby preserving cell viability for complementary downstream analyses.

Using this UHF-DEP–based workflow, we demonstrated that cells with high TMB could be distinguished from low-TMB cells using an EMS median frequency. TMB classification was defined using Oncomine™ data (≥10 Mut/Mb) consistent with FDA-approved thresholds, without requiring the companion diagnostic from FMI.

Importantly, EMS correlated significantly with TMB across diverse solid tumor cell lines, reinforcing the biological relevance of this parameter. However, insights from experiments involving the mutagenic agent N-ethyl-N-nitrosourea (ENU) highlight certain limitations. ENU induces random, non-physiological mutations that do not mirror the genetic evolution of oncogenesis. Consequently, the observed increases in EMS following ENU exposure may reflect cumulative genetic instability rather than clinically relevant mutational burden. This distinction suggests that mutational load, expressed as variant count, might better represent the degree of divergence between the parental and transformed cell populations, rather than an absolute index of mutation frequency. Further studies using more physiologically relevant models, such as oncogene-driven transformation or primary tumor cells, are warranted to validate this hypothesis. We acknowledge that our proof-of-concept study is limited by the number of independent biological replicates, and that formal power analyses to discriminate closely related cell lines would require substantially larger datasets. Consequently, our primary aim was to evaluate whether UHF-DEP–derived parameters capture biologically meaningful trends rather than to establish definitive classification thresholds. The similarity observed between certain cell line profiles highlights the limited discriminative power of EMS when used in isolation, reinforcing that comparisons with healthy counterparts are intended to contextualize trends rather than to achieve perfect classification.

Clinically, these observations imply that the most meaningful application of EMS may rely on comparative analyses between tumor cells and their matched healthy counterparts. Such differential assessments could provide a personalized dielectric reference, enhancing the sensitivity and interpretability of EMS-based diagnostics.

Overall, our findings support the potential of UHF-DEP analysis as a complementary tool for biomarker assessment, particularly for mutations captured through TMB. By enabling the detection of biophysical variations associated with mutational load, UHF-DEP could enhance patient stratification for ICI therapy and contribute to more personalized treatment selection.

These preliminary data underscore the potential of UHF-DEP to characterize cancer cells, paving the way for its broader application in patient stratification. Future research should focus on establishing standardized operating protocols, evaluating inter-sample reproducibility, and benchmarking UHF-DEP performance against established molecular assays and biomarkers. Furthermore, integrating UHF-DEP with sequencing-based tests could improve predictive accuracy by enabling multi-parametric biomarker strategies, with UHF-DEP serving as a complementary biophysical indicator rather than as a sole classification criterion. Large-scale studies across diverse cancer types will be essential to confirm that patients identified using this approach derive tangible clinical benefit from ICIs, validating UHF-DEP as a predictive and clinically actionable biomarker.

## Figures and Tables

**Table 1:** Validation of Tumor Mutational Burden (TMB) and mutation counts was performed using Oncomine, with comparisons to Foundation Medicine (FMI) data and Cancer Cell Line Encyclopedia (CCLE) data from the Broad Institute. (**A**) TMB values and mutation counts for various cell lines (U87-MG, DAOY, H1975, MEL-28, MEL-5, A549, and SW480) were obtained using both Oncomine and CCLE platforms. The CCLE data from the Broad Institute served as a reference to ensure consistency with the Oncomine results. TMB values and mutation counts are presented for each cell line. (**B**) TMB values for negative template (Neg. template), Tumor A and Tumor B, obtained using the Oncomine kit, are compared with FMI data for the same tumors, which were used as the gold standard for validation. The negative control template is also shown.

(A)					(B)		
	Oncomine^TM^		CCLE			TMB	
	TMB	Mutation Count	TMB	Mutation Count		Foundation Medicine	Oncomine^TM^
U87-MG	3.69	613	4.77	140	Neg. template		0
DAOY	5.9	804	7.27	214	Tumor A	0	1.78
H1975	9.56	707	11.36	337	Tumor B	117	90.18
MEL-28	11.66	739	10.67	321			
MEL-5	13.45	966	18.57	549			
A549	14.76	859	18.97	561			
SW480	17.97	709	18.8	565			

## Data Availability

The original contributions presented in this study are included in the article/Supplementary Materials. The raw data supporting the conclusions of this article will be made available by the authors on request.

## Supplementary Materials

The following supporting information can be downloaded at: https://www.mdpi.com/article/10.3390/bios16030134/s1, Figure S1: Example of reproducibility of biological replicates assessed by UHF-DEP analysis; Supplementary Materiel and Method S1: Dead cell removal.

## Abbreviations

The following abbreviations are used in this manuscript:

ATCC	American Type Culture Collection
CCLE	Cancer Cell Line Encyclopedia
CF	Crossover Frequency
CTLA-A	Cytotoxic T-lymphocyte-associated protein 4
DEP	Dielectrophoresis
DEP-B	DEP-Buffer
DMEM	Dulbecco’s Modified Eagle Medium
DMSO	Dimethyl Sulfoxide
DNA	Deoxyribonucleic Acid
EMS	Electromagnetic Signature
ENU	N-ethyl-N-nitrosourea
FDA	Food and Drug Administration
FBS	Fetal Bovine Serum
FDEP	Dielectrophoretic Force
FMI	Foundation Medicine, Inc.
ICI	Immune Checkpoint Inhibitors
nDEP	negative DEP
pDEP	Positive DEP
PCR	Polymerase Chain Reaction
qPCR	Quantitative Polymerase Chain Reaction
PD-1	Programmed cell Death protein 1
PD-L1	Programmed Death-Ligand 1
RF	Radio Frequency
TMB	Tumor Mutational Burden
UHF	Ultra High-Frequency
VSWR	Voltage Standing Wave Ratio
WES	Whole Exome Sequencing

## References

[1] Tan S., Li D., Zhu X. (2020). Cancer immunotherapy: Pros, cons and beyond. Biomed. Pharmacother..

[2] Shiravand Y., Khodadadi F., Kashani S.M.A., Hosseini-Fard S.R., Hosseini S., Sadeghirad H., Ladwa R., O’Byrne K., Kulasinghe A. (2022). Immune Checkpoint Inhibitors in Cancer Therapy. Curr. Oncol..

[3] Salik B., Smyth M.J., Nakamura K. (2020). Targeting immune checkpoints in hematological malignancies. J. Hematol. Oncol..

[4] Klein O., Kee D., Markman B., Carlino M.S., Underhill C., Palmer J., Power D., Cebon J., Behren A. (2021). Evaluation of TMB as a predictive biomarker in patients with solid cancers treated with anti-PD-1/CTLA-4 combination immunotherapy. Cancer Cell.

[5] Butterfield L.H., Najjar Y.G. (2024). Immunotherapy combination approaches: Mechanisms, biomarkers and clinical observations. Nat. Rev. Immunol..

[6] Chen X.-J., Yuan S.-Q., Duan J.-L., Chen Y.-M., Chen S., Wang Y., Li Y.-F. (2020). The Value of PD-L1 Expression in Predicting the Efficacy of Anti-PD-1 or Anti-PD-L1 Therapy in Patients with Cancer: A Systematic Review and Meta-Analysis. Dis. Markers.

[7] Garon E.B., Rizvi N.A., Hui R., Leighl N., Balmanoukian A.S., Eder J.P., Patnaik A., Aggarwal C., Gubens M., Horn L. (2015). Pembrolizumab for the Treatment of Non–Small-Cell Lung Cancer. N. Engl. J. Med..

[8] Socinski M.A., Jotte R.M., Cappuzzo F., Orlandi F., Stroyakovskiy D., Nogami N., Rodríguez-Abreu D., Moro-Sibilot D., Thomas C.A., Barlesi F. (2018). Atezolizumab for First-Line Treatment of Metastatic Nonsquamous NSCLC. N. Engl. J. Med..

[9] Morihiro T., Kuroda S., Kanaya N., Kakiuchi Y., Kubota T., Aoyama K., Tanaka T., Kikuchi S., Nagasaka T., Nishizaki M. (2019). PD-L1 expression combined with microsatellite instability/CD8+ tumor infiltrating lymphocytes as a useful prognostic biomarker in gastric cancer. Sci. Rep..

[10] Mok T.S.K., Wu Y.-L., Kudaba I., Kowalski D.M., Cho B.C., Turna H.Z., Castro G., Srimuninnimit V., Laktionov K.K., Bondarenko I. (2019). Pembrolizumab versus chemotherapy for previously untreated, PD-L1-expressing, locally advanced or metastatic non-small-cell lung cancer (KEYNOTE-042): A randomised, open-label, controlled, phase 3 trial. Lancet.

[11] Wakelee H., Liberman M., Kato T., Tsuboi M., Lee S.-H., Gao S., Chen K.-N., Dooms C., Majem M., Eigendorff E. (2023). Perioperative Pembrolizumab for Early-Stage Non–Small-Cell Lung Cancer. N. Engl. J. Med..

[12] Berghmans T., Durieux V., Hendriks L.E.L., Dingemans A.-M. (2020). Immunotherapy: From Advanced NSCLC to Early Stages, an Evolving Concept. Front. Med..

[13] Chen D.S., Mellman I. (2017). Elements of cancer immunity and the cancer–immune set point. Nature.

[14] Makuku R., Khalili N., Razi S., Keshavarz-Fathi M., Rezaei N. (2021). Current and Future Perspectives of PD-1/PDL-1 Blockade in Cancer Immunotherapy. J. Immunol. Res..

[15] Chalmers Z.R., Connelly C.F., Fabrizio D., Gay L., Ali S.M., Ennis R., Schrock A., Campbell B., Shlien A., Chmielecki J. (2017). Analysis of 100,000 human cancer genomes reveals the landscape of tumor mutational burden. Genome Med..

[16] Choucair K., Morand S., Stanbery L., Edelman G., Dworkin L., Nemunaitis J. (2020). TMB: A promising immune-response biomarker, and potential spearhead in advancing targeted therapy trials. Cancer Gene Ther..

[17] Jardim D.L., Goodman A., De Melo Gagliato D., Kurzrock R. (2021). The Challenges of Tumor Mutational Burden as an Immunotherapy Biomarker. Cancer Cell.

[18] Aggarwal C., Ben-Shachar R., Gao Y., Hyun S.W., Rivers Z., Epstein C., Kaneva K., Sangli C., Nimeiri H., Patel J. (2023). Assessment of Tumor Mutational Burden and Outcomes in Patients with Diverse Advanced Cancers Treated With Immunotherapy. JAMA Netw. Open.

[19] Hellmann M.D., Ciuleanu T.-E., Pluzanski A., Lee J.S., Otterson G.A., Audigier-Valette C., Minenza E., Linardou H., Burgers S., Salman P. (2018). Nivolumab plus Ipilimumab in Lung Cancer with a High Tumor Mutational Burden. N. Engl. J. Med..

[20] Marabelle A., Fakih M., Lopez J., Shah M., Shapira-Frommer R., Nakagawa K., Chung H.C., Kindler H.L., Lopez-Martin J.A., Miller W.H. (2020). Association of tumour mutational burden with outcomes in patients with advanced solid tumours treated with pembrolizumab: Prospective biomarker analysis of the multicohort, open-label, phase 2 KEYNOTE-158 study. Lancet Oncol..

[21] Litchfield K., Reading J.L., Puttick C., Thakkar K., Abbosh C., Bentham R., Watkins T.B.K., Rosenthal R., Biswas D., Rowan A. (2021). Meta-analysis of tumor- and T cell-intrinsic mechanisms of sensitization to checkpoint inhibition. Cell.

[22] Gutierrez M., Lam W.-S., Hellmann M.D., Gubens M.A., Aggarwal C., Tan D.S.W., Felip E., Chiu J.W.Y., Lee J.-S., Yang J.C.-H. (2023). Biomarker-directed, pembrolizumab-based combination therapy in non-small cell lung cancer: Phase 2 KEYNOTE-495/KeyImPaCT trial interim results. Nat. Med..

[23] Xie N., Shen G., Gao W., Huang Z., Huang C., Fu L. (2023). Neoantigens: Promising targets for cancer therapy. Signal Transduct. Target. Ther..

[24] Sha D., Jin Z., Budczies J., Kluck K., Stenzinger A., Sinicrope F.A. (2020). Tumor Mutational Burden as a Predictive Biomarker in Solid Tumors. Cancer Discov..

[25] Duncan J.L., Bloomfield M., Swami N., Cimini D., Davalos R.V. (2023). High-Frequency Dielectrophoresis Reveals That Distinct Bio-Electric Signatures of Colorectal Cancer Cells Depend on Ploidy and Nuclear Volume. Micromachines.

[26] Pethig R. (2017). Dielectrophoresis: Theory, Methodology and Biological Applications.

[27] Afshar S., Fazelkhah A., Braasch K., Salimi E., Butler M., Thomson D.J., Bridges G.E. (2021). Full Beta-Dispersion Region Dielectric Spectra and Dielectric Models of Viable and Non-Viable CHO Cells. IEEE J. Electromagn. RF Microw. Med. Biol..

[28] Lambert E., Manczak R., Barthout E., Saada S., Porcù E., Maule F., Bessette B., Viola G., Persano L., Dalmay C. (2021). Microfluidic Lab-on-a-Chip Based on UHF-Dielectrophoresis for Stemness Phenotype Characterization and Discrimination among Glioblastoma Cells. Biosensors.

[29] Manczak R., Saada S., Provent T., Dalmay C., Bessette B., Bégaud G., Battu S., Blondy P., Jauberteau M., Kaynak C.B. (2019). UHF-Dielectrophoresis Crossover Frequency as a New Marker for Discrimination of Glioblastoma Undifferentiated Cells. IEEE J. Electromagn. RF Microw. Med. Biol..

[30] Hanahan D., Weinberg R.A. (2011). Hallmarks of Cancer: The Next Generation. Cell.

[31] Hjeij F., Dalmay C., Bessaudou A., Blondy P., Pothier A., Bessette B., Begaud G., Jauberteau M.O., Lalloue F., Kaynak C.B. (2016). UHF dielectrophoretic handling of individual biological cells using BiCMOS microfluidic RF-sensors. Proceedings of the 2016 46th European Microwave Conference (EuMC).

[32] Hyler A.R., Hong D., Davalos R.V., Swami N.S., Schmelz E.M. (2021). A novel ultralow conductivity electromanipulation buffer improves cell viability and enhances dielectrophoretic consistency. Electrophoresis.

[33] Ghandi M., Huang F.W., Jané-Valbuena J., Kryukov G.V., Lo C.C., McDonald E.R., Barretina J., Gelfand E.T., Bielski C.M., Li H. (2019). Next-generation characterization of the Cancer Cell Line Encyclopedia. Nature.

[34] Cerami E., Gao J., Dogrusoz U., Gross B.E., Sumer S.O., Aksoy B.A., Jacobsen A., Byrne C.J., Heuer M.L., Larsson E. (2012). The cBio Cancer Genomics Portal: An Open Platform for Exploring Multidimensional Cancer Genomics Data. Cancer Discov..

[35] Rui R., Zhou L., He S. (2023). Cancer immunotherapies: Advances and bottlenecks. Front. Immunol..

[36] Budczies J., Kazdal D., Menzel M., Beck S., Kluck K., Altbürger C., Schwab C., Allgäuer M., Ahadova A., Kloor M. (2024). Tumour mutational burden: Clinical utility, challenges and emerging improvements. Nat. Rev. Clin. Oncol..

[37] Hanahan D. (2022). Hallmarks of Cancer: New Dimensions. Cancer Discov..

