# Supplementary material for: Proof of Concept for Tumor Mutational Burden Prediction Through Biophysical Analysis Based on UHF-Dielectrophoresis
Source: Biosensors (Basel). 2026 Feb 25;16(3):134. doi: 10.3390/bios16030134 (PMC13023853; doi:10.3390/bios16030134)
Supplement: Supplementary file 1 [file biosensors-16-00134-s001.zip › biosensors-4114353-supplementary.pdf]

biosensors

Supplementary Materials

Proof of Concept for Tumor Mutational Burden Prediction through Biophysical Analysis based on UHF-Dielectrophoresis

Héloïse Daverat [1,†], Nina Blasco [2,†], Sandrine Robert [1], Amandine Rovini [1,3], Claire Dalmay [2], Fabrice Lalloué [1,3], Arnaud Pothier [2,‡], Karine Durand [1,4,5,*,‡] and Thomas Naves [1,3,*,‡]

[1] UMR INSERM 1308, CAPTuR, University of Limoges, 2 Rue du Docteur Marcland, 87025 Limoges, France; heloise.daverat@unilim.fr (H.D.); sandrine.robert@unilim.fr (S.R.); amandine.rovini@unilim.fr (A.R.); fabrice.lalloue@unilim.fr (F.L.)
[2] XLIM-UMR 7252, University of Limoges/CNRS, 123 Avenue Albert Thomas, 87060 Limoges, France; nina.blasco@xlim.fr (N.B.); claire.dalmay@xlim.fr (C.D.); arnaud.pothier@xlim.fr (A.P.)
[3] Chaire of Experimental Pneumology Biorescan, University of Limoges-Partnership Foundation, 2 Rue du Docteur Marcland, 87025 Limoges, France
[4] Molecular Genetics Platform of Solid Cancers, Department of Pathological Anatomy, Limoges University Hospital, 2 Avenue Martin Luther King, 87042 Limoges, France
[5] Functional Unit of Support to Translational Research and Innovation in Solid Oncology, Limoges University Hospital, 2 Avenue Martin Luther King, 87042 Limoges, France
* Correspondence: karine.durand@chu-limoges.fr (K.D.); thomas.naves@unilim.fr (T.N.); Tel.: +33-555-435-970 (T.N.)
† These authors contributed equally to this work.
‡ These authors contributed equally to this work.

Supplementary Figure S1

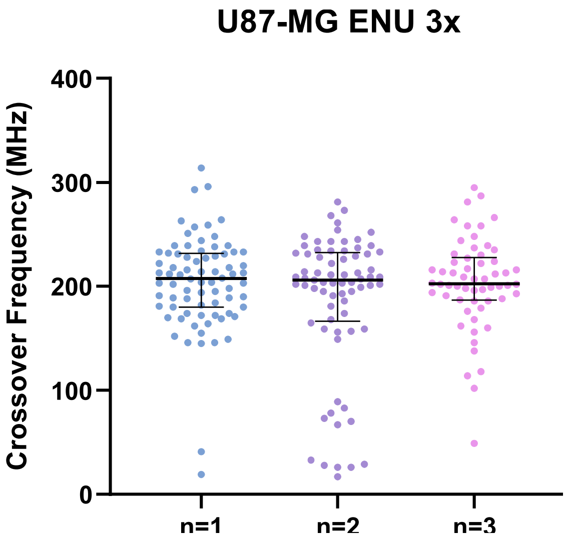

Figure S1. Example of reproducibility of biological replicates assessed by UHF-DEP analysis.

The scatter plot illustrats the reproducibility of biological replicates in UHF-DEP analysis across three experimental conditions with U87-MG ENU 3x. Each dot represents an individual crossover frequency measurement from a single cell, while each “n” refers to an independent biological replicate (n = 3). The total number of cells analyzed was 76, 73, and 58 for replicates 1, 2, and 3, respectively. The crossover frequency value medians (n=1: 207.5 MHz, n=2: 206.0 MHz, n=3: 202.5 MHz) and interquartile ranges (IQR) are shown for each replicate. These results demonstrate consistent and comparable measurements across biological replicates, supporting the robustness and reproducibility of the

method. No significant differences were observed by using a Kruskal–Wallis test applied to the median crossover frequency values of the three independent biological replicates.

Supplementary Material end Method S1

Dead Cell Removal

Live cell sorting was performed using the Dead Cell Removal Kit (Miltenyi Biotec, Germany) and MS Columns (Miltenyi Biotec), following the supplier's recommendations. Cell viability was systematically checked before and after sorting using the LUNA-II™ automated cell counter (Logos Biosystems, South Korea) and trypan blue staining (Sigma-Aldrich).

https://doi.org/10.3390/bios16030134
